# CTR9, a Component of PAF Complex, Controls Elongation Block at the *c-Fos* Locus via Signal-Dependent Regulation of Chromatin-Bound NELF Dissociation

**DOI:** 10.1371/journal.pone.0061055

**Published:** 2013-04-11

**Authors:** Hyun-Seok Yoo, Jung-Hwa Seo, Joo-Yeon Yoo

**Affiliations:** Division of Molecular and Life Sciences, Department of Life Sciences, Pohang University of Science and Technology (POSTECH), Pohang, Republic of Korea; Korea University, Republic of Korea

## Abstract

PAF complex (PAFc) is an RNA polymerase II associated factor that controls diverse steps of transcription. Although it is generally associated with actively transcribed genes, a repressive PAFc has also been suggested. Here, we report that PAFc regulates the transition from transcription initiation to transcription elongation. PAFc repressed IL-6-induced, but not TNF-α-induced, immediate early gene expression. PAFc constitutively associated with the 5′-coding region of the c-Fos locus, then transiently dissociated upon IL-6 stimulation. When CTR9, a component of PAFc, was depleted, higher levels of serine 5-phosphorylated or serine 2-phosphorylated forms of RNA Polymerase II were associated with the unstimulated c-Fos locus. We also observed an increased association of CDK9, a kinase component of the pTEF-b elongation factor, with the c-Fos locus in the CTR9-depleted condition. Furthermore, association of negative elongation factor, NELF, which is required to proceed to the elongation phase, was significantly reduced by CTR9 depletion, whereas elongation factor SPT5 recruitment was enhanced by CTR9 depletion. Finally, the chromatin association of CTR9 was specifically controlled by IL-6-induced kinase activity, because a JAK2 kinase inhibitor, AG-490, blocked its association. In conclusion, our data suggest that PAFc controls the recruitment of NELF and SPT5 to target loci in a signal- and locus-specific manner.

## Introduction

Transcription occurs in three phases: initiation, elongation, and termination. General transcription factors recognize promoter sequences and recruit RNA polymerase II (Pol II) to form a preinitiation complex (PIC), the first step of messenger RNA (mRNA) synthesis [1]. Before proceeding to the elongation phase, the functional and structural block created by the pre-initiation complex (PIC), a complex process that is regulated by multiple elongation factors, has to be cleared [2]. Appropriate cellular responses to specific stimuli depend on signaling cascades to activate transcription factors (TFs) that bind to specific gene regulatory regions. Generally, TF binding facilitates chromatin remodeling and the recruitment of eukaryotic RNA Pol II to the transcription start site (TSS) to initiate transcription [3]. However, a genome-wide analysis of eukaryotic RNA Pol II revealed that over 30% of human genes are blocked from proceeding to elongation by an RNA Pol II PIC at the promoter-proximal region [4].

Among these blocked genes are the immediate early genes (IEGs), such as *c-Fos*, *junB*, and *c-myc*, which are rapidly induced by external stimuli [5]–[7]. In contrast to the stress-responsive genes whose transcription is tightly controlled by signal-specific TFs at the pre-initiation step, the promoters of IEGs are pre-occupied by RNA Pol II to facilitate rapid induction, and the signal-induced transcription of IEGs is primarily controlled at the transition from initiation into elongation [8]–[10]. Therefore, the transcriptional machinery has diverse mechanisms to ensure the efficiency and specificity of gene regulation.

While moving through the template DNA, eukaryotic RNA Pol II encounters nucleosomes. The concerted actions of histone modifying enzymes, ATP-dependent chromatin remodelers, and histone chaperones are necessary for the efficient movement of RNA Pol II through nucleosomes [11]. Stepwise transcriptional processes and co-transcriptional pre-mRNA processing require a structural platform to ensure the coordinated regulation of transcription. The C-terminal domain (CTD) of RNA Pol II serves as a major assembly point for the binding of multiple proteins that control transcription, RNA processing, and histone modification [12]. Both genetic and biochemical approaches have been applied to identify the RNA Pol II-associated transcription factors that regulate these processes. Among these transcriptional regulators is the PAFc, which was originally identified in yeast.

PAFc is an evolutionarily conserved RNA Pol II-associated complex that is composed of Paf1, Ctr9, Cdc73, Rtf1, and Leo1 in yeast [13]–[16]. In humans, PAFc contains an additional subunit, SKI8 [17]. PAFc is involved in multiple steps of transcription, including initiation, elongation and mRNA 3′-end-processing [18]. PAFc associates with the chromatin of actively transcribing genes and plays critical roles in the recruitment of the histone modifying machinery to target loci [19], [20]. Furthermore, PAFc interacts with the FACT complex and hDst1/SII, and stimulates transcriptional elongation *in vitro* [21], [22]. Because PAFc associates with chromatin and physically interact with multiple transcriptional regulators, most work on PAFc has primarily focused on its role in the positive regulation of transcription. However, accumulating evidence indicates that there are groups of genes that are negatively regulated by PAFc [23]–[25], although the molecular mechanisms governing its negative effect are not well understood.

Previously, we reported that PAFc participates in the transcriptional activation of acute phase protein (APP) genes through the direct interaction of CTR9 and STAT3 [26]. In a search for general mechanisms of target gene selection by PAFc, we found that PAFc behaves as a dual regulator; it acts as a transcriptional activator for stimulus-dependent APP gene induction, whereas the transcription of IEGs is negatively controlled by PAFc. In this study, the molecular mechanism governing PAFc-mediated negative regulation of *c-Fos* transcription was explored.

## Materials and Methods

### Plasmids

A full-length cDNA of mouse Ctr9 was obtained in the laboratory of S. Desiderio, as previously described [27]. Mouse Paf1 cDNA was obtained by PCR and cloned into pCDNA3.1 myc-His(A) vector (Invitrogen, Clarlsbad, CA). A 2.0 kb promoter fragment of *c-Fos* gene, 1.3 kb fragment containing 0.4 kb promoter and 1.1 kb coding region of *c-Fos*, and a total 3.5 kb *c-Fos* genomic region fragment were obtained by PCR with appropriate primer sets and cloned into pGL3 basic vector (Promega, Madison, WI). 0.9 kb *c-Fos* first intron was obtained by PCR with appropriate primer sets and cloned into pGL3 control vector (Promega, Madison, WI). The sequences of the primers for PCR are provided in Table S1.

### Cell Culture

HepG2 cells were maintained in MEM (Welgene, Korea) supplemented with 10% fetal bovine serum (Hyclone, Logan, UT) and maintained at 37°C in a 5% CO_2_ atmosphere. For cytokine stimulation, cells were treated with rhIL-6 (20 ng/ml) plus IL-6sR (20 ng/ml) for indicated times (R&D Systems, Minneapolis, MN). Transient transfection was carried out using Lipofectamine 2000 (Invitrogen, Clarlsbad, CA) according to the manufacturer's instructions.

### RNA interference

Oligomer sequences used for RNA interference are as follows; Control: 5′-UUCUCCGAACGUGUCACGUTT-3′, CTR9: 5′-CCGUGUGGCUCCAAACUUUATT-3′, LEO1: 5′-GCCGGUAGCUUCUGAUAAUTT-3′, CDC73: 5′- GGUACAUGGUAAAGCAUAATT-3′, PAF1: 5′-GCAG UUUACCGAGGAAGAATT-3′, NELF-E: 5′-GACCCAGAUUGUCUACAG UTT-3′, SPT5: 5′-GGACUGUCAAAUGUAAGAUTT-3′.

#### RNA Preparation and Analysis

Total RNA was extracted from cells using the RNAiso Plus (Takara Bio, Shiga, Japan). For conventional or real time PCR, 1 µg of total RNA was reverse-transcribed using oligo-d (T)_15_ primer or random hexamer (Promega, Madison, WI), respectively, and M-MLV reverse transcriptase (Promega, Madison, WI). For the Nuclear/Cytoplasmic fractionation, PARIS kit (Ambion, Austin, TX) was used, according to the manufacturer's instructions. The sequences of the primers for PCR are provided in Table S1. Presented data are representatives of more than two biological replicates and indicated error bars came from three technical replicates.

### Chromatin Immunoprecipitation (ChIP)

ChIP was performed as previously described [26]. Antibodies against CDK9, NELF-E, Pol II N-terminal, and SPT5 were purchased from Santa Cruz Biotechnology (Santa Cruz, CA). Also, antibodies against Serine 5P and Serine 2P Pol II were purchased from Abcam (Cambridge, MA). The sequences of the primers for PCR are provided in Table S1. Presented data are representatives of more than two biological replicates and indicated error bars came from three technical replicates.

### Immunoblotting and Immunoprecipitation

HepG2 cells were lysed in lysis buffer (25 mM Tris, pH 7.5, 150 mM NaCl, 1% Triton X-100, 0.5% deoxycholic acid, 0.1% SDS, 0.1 mg/ml PMSF, 1 mM DTT, 1 mM sodium orthovanadate, 5 µg/ml aprotinin, 2 µg/ml pepstatin, 5 µg/ml leupeptin, 1 mM benzamidine). Total cell lysates (40 µg per sample) were resolved using SDS-polyacrylamide gels. For immunoprecipitation, 1 mg of lysate was incubated with 2 ug of antibody overnight. NaF (25 mM), β-glycerol phosphate (25 mM) and Na_3_VO_4_ (5 mM) were added additionally to detect phosphorylated forms of RNA polymerase II during the immunoprecipitation. Antibodies against CTR9, PAF1, CDC73, and LEO1 were purchased from Bethyl Laboratory (Montgomery, TX). Antibodies against STAT3, phospho-STAT3(Y705), TBP, c-Myc and NELF-E were purchased from Santa Cruz Biotechnology. Antibody against GAPDH was purchased from Chemicon (Chemicon International, Temecula, CA).

### Fractionation

Chromatin-bound and –unbound fractions were prepared as previously described [28]. Briefly, HepG2 cells were incubated on ice for 5 min with 100 mM NaCl, 300 mM sucrose, 3 mM MgCl_2_, 10 mM PIPES (pH 6.8), 1 mM EGTA, 0.2% Triton X-100, and protease inhibitors (0.1 mg/ml PMSF, 1 mM DTT, 1 mM sodium orthovanadate, 5 µg/ml aprotinin, 2 µg/ml pepstatin, 5 µg/ml leupeptin, 1 mM benzamidine). Cells were then centrifugated, and the supernatant was collected as the “unbound” fraction. The remaining pellet was further incubated with 0.5 U/µl DNaseI containing buffer 37°c for 30 min, centrifugated, and the supernatant was collected as the “bound” fraction.

## Results

### c-Fos transcription is negatively regulated by PAFc in the nucleus

**Figure 1:**
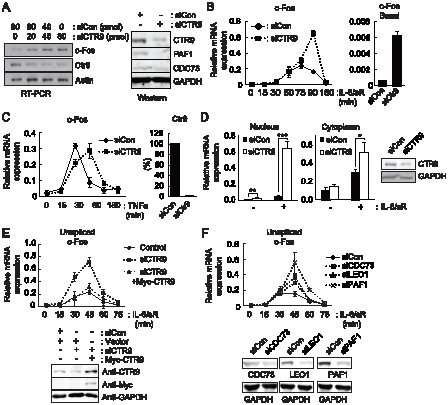
c-Fos transcription is negatively regulated by PAFc in the nucleus. *A*, Left, transcript levels of c-Fos, Ctr9 and Actin were analyzed by RT-PCR in HepG2 cells transfected with control siRNA (siCon) or CTR9 siRNA (siCTR9), as indicated above. Right, endogenous protein levels of PAFc components in CTR9 knockdown cells were measured by western blot analysis. *B*, IL-6-induced and basal c-Fos expression was analyzed by Real-time RT-PCR (RT-qPCR). HepG2 cells were transfected with either control or CTR9 siRNA. Approximately 48 hours later, cells were treated with IL-6 plus IL-6sR (20 ng/ml each) for the indicated length of time. Expression levels were normalized to β-Actin. *C*, Cells were transfected with Control or CTR9 siRNA. 48 hours later, cells were treated with TNFα (20 ng/ml) for the indicated length of time. *D*, Left, Nuclear and cytoplasmic RNA were prepared using the PARIS kit. The levels of c-Fos mRNA in the nuclear and cytoplasmic fractions were then analyzed by RT-qPCR. Right, endogenous protein levels of CTR9 in control and knockdown cells were measured by western blot analysis. *E*, Cells were transfected as indicated. 48 hours later, cells were treated with IL-6 plus IL-6sR (20 ng/ml each) for the indicated length of time. The unspliced c-Fos transcripts were analyzed by RT-qPCR. Bottom, endogenous protein levels of CTR9 and exogenous Myc-CTR9 were measured by western blot analysis. *F*, PAFc components were individually knocked down by transient siRNA transfection. Samples were prepared and analyzed as described in *E*. Bottom, endogenous protein levels of PAFc components were measured by western blot analysis. **p<0.01, ***p<0.001 by Student's t test. Error bars represents standard deviation(SD) (n = 3).

We have previously shown that CTR9, a component of PAFc, aids the transcription of IL-6-inducible acute phase protein (APP) genes, such as *fibrinogen* (*FGG*) and *haptoglobin* (*Hp*), by stabilizing the binding between STAT3 and chromatin at promoters [26]. This finding prompted us to investigate whether CTR9 aids the transcription of all genes whose transcription is mediated by STAT3. We studied an immediate early gene, *c-Fos*, which is transcriptionally induced by IL-6 in hepatocytes. The proto-oncogene *c-Fos* contains a canonical STAT3 binding site in its promoter region and is known to be directly controlled by IL-6-activated STAT3 [29]. Surprisingly, we found that knockdown of endogenous CTR9 led to a marked increase in *c-Fos* transcription under both basal and IL-6-induced conditions (Fig. 1*A and 1B*). As previously reported, reduced CTR9 expression led to a decrease in the expression of other components of PAFc (Fig. 1*A*) [17]. To assess whether the negative effect of PAFc on *c-Fos* transcription is IL-6-signal specific, we then stimulated cells with TNFα, which induces *c-Fos* expression through activation of the NFκB signaling pathway (Fig. 1*C*). In contrast to the effect of IL-6 stimulation, TNFα-mediated induction of *c-Fos* was not significantly enhanced in the CTR9-deficient cells. This demonstrates that the negative effect of PAFc on *c-Fos* transcription occurs in a signal-specific manner.

PAFc is known to be involved in multiple transcriptional steps, including initiation, elongation, and the 3′-end processing of mRNA [18]. Because most of these regulatory processes occur in the nucleus, we harvested mRNAs from the nucleus and the cytoplasm separately and assessed the regulatory effect of CTR9 knockdown on each of these mRNA populations (Fig. 1*D*). Although a negative effect of CTR9 was observed in the *c-Fos* mRNA prepared from the cytoplasmic fraction, the most dramatic effect was observed in the RNA prepared from the nuclear fraction. The knockdown effect of CTR9 on *c-Fos* transcription was observed in unspliced transcripts, and overexpression of mouse CTR9, which is insensitive to human CTR9 siRNA, successfully reversed the hyper-induction of *c-Fos* in the CTR9 knockdown condition (Fig. 1*E*). However, Myc-CTR9 overexpression alone did not alter the expression of *c-Fos* significantly (Fig. S1 *A*). Since CTR9 is relatively abundant than other PAFc components in yeast [30], it is possible that endogenous CTR9 is already saturated to exhibit inhibitory effect. At the same time, it is also possible that CTR9 requires other protein for its inhibitory action. Since CTR9 is reported to act as a component of PAFc (PAFc) [15], [31]–[33], we next examined the contribution of other components of PAFc on *c-Fos* transcription (Fig. 1*F*). Although the effect was not as strong as CTR9, unspliced *c-Fos* transcripts were increased by individual knockdown of CDC73, LEO1 or PAF1. When individual component of PAFc such as PAF1 or CDC73 was overexpressed, it did not significantly changed *c-Fos* expression (Fig. S1 *B*). These data demonstrate that PAFc plays a negative role in the transcriptional control of *c-Fos* in the nucleus.

### PAFc controls the transition from the initiation to elongation phases of transcription at the c-Fos locus

During the transcriptional control of *c-Fos*, the elongation step is critical [34]. The promoter-proximal region of *c-Fos* is pre-occupied by RNA Pol II to form a pre-initiation complex that is stalled by an elongation block present in the first intron of *c-Fos* [35]. The stalled RNA Pol II only enters the elongation phase after stimuli trigger the recruitment of positive elongation factor b (pTEF-b) to the phosphorylated CTD of RNA Pol II; pTEF-b then recruits other elongation factors [36]. Because PAFc is known to associate with elongation factors, such as pTEF-b, SPT4/5, and the FACT complex, and aids RNA Pol II elongation *in vitro* [16], [21], we examined its effect on the control of elongation during *c-Fos* transcription.

**Figure 2:**
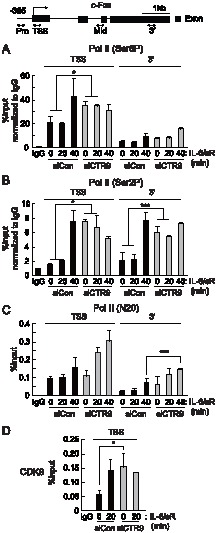
Elongating RNA Pol II pre-occupies the c-Fos locus in the CTR9-deficient cells. Top, diagram of the c-Fos genomic loci. Every PCR primers used for ChIP analyses in this study are shown as two-sided arrows. HepG2 cells were transfected with control siRNAs or CTR9 siRNAs and treated with IL-6 plus IL-6sR (20 ng/ml each) for the indicated length of time. ChIP analyses were performed with soluble chromatin using antibodies as indicated: anti-Ser5P CTD (*A*), anti-Ser2P CTD (*B*), anti-RNA polymerase II N-terminus (*C*), and anti-CDK9 (*D*),. Bound DNA was analyzed by quantitative PCR using primers specific to c-Fos loci. *p<0.05, ***p<0.001 by Student's t test. Error bars represents SD (n = 3).

The association of modified RNA Pol II with chromatin at the *c-Fos* genomic locus was first examined by ChIP analysis using antibodies that detect the serine 5-phosphorylated or serine 2-phosphorylated forms of RNA Pol II. RNA Pol II phosphorylated on the serine 5 residue of the CTD (Ser5P RNA Pol II) was mainly detected in the TSS region of *c-Fos* under basal conditions, and this association was further enhanced by IL-6 stimulation (Fig. 2*A*). In CTR9-depleted cells, slightly higher levels of Ser5P RNA Pol II were found in the TSS region of *c-Fos* under basal conditions, indicating that CTR9 plays negative roles in the progression of transcription under basal conditions. Even more interestingly, the CTD of RNA Pol II associated with the coding regions of *c-Fos* was heavily phosphorylated on serine 2 residues even in the absence of IL-6 stimulation and remained high after IL-6 stimulation (Fig. 2*B*). Upon IL-6 stimulation, levels of the phosphorylated serine 5 or serine 2 in the CTR9-depleted cells were either statistically increased or not changed, compared to control cells. Consistent with its role in transcription elongation, more RNA Pol II was found in the 3′- coding region of the *c-Fos* locus in the CTR9-deficient cells (Fig. 2*C*). These results indicate that when CTR9 is limited, stalled RNA Pol II at the *c-Fos* locus can proceed to the elongation phase without stimulation. In other words, the association of PAFc with *c-Fos* functions to prevent elongation; dissociation of PAFc from the coding region of *c-Fos* might therefore be required for transcriptional elongation.

To determine the role of PAFc in the process of elongation, we examined the recruitment of pTEF-b to the *c-Fos* locus upon stimulation. At basal condition, weak association of CDK9, a kinase component of pTEF-b, was detected, and this association was further increased after IL-6 stimulation (Fig. 2*D*). When CTR9 was depleted, CDK9 association with *c-Fos* TSS region increased significantly, even in the absence of stimulation (Fig. 2*D*). Therefore, it is possible that the enhanced phosphorylation of the serine 5 and serine 2 residues of RNA Pol II CTD in the absence of CTR9 might be partially resulted from the increased CDK9 association to the *c-Fos* locus. These results collectively indicate that the RNA Pol II transition from the initiation phase to the elongation phase was significantly increased at the *c-Fos* locus in the absence of CTR9.

### Recruitment of the negative elongation factor NELF to the c-Fos locus requires CTR9

**Figure 3:**
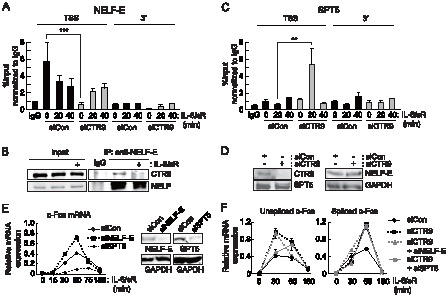
Recruitment of the negative elongation factor NELF to the c-Fos locus requires CTR9. Cells were transfected with control or CTR9 siRNA and treated with IL-6 and IL-6sR (20 ng/ml each) for the indicated length of time. ChIP analyses were performed with soluble chromatin using the antibodies indicated, and the bound DNA was analyzed by quantitative PCR using primers specific to c-Fos. Antibodies specific to NELF-E (*A*), or SPT5 (*C*), were used. *B*, Cells were treated with IL-6 and IL-6sR (20 ng/ml each) for 30 minutes and lysates were immunoprecipitated with anti-NELF-E antibody. Immunoprecipitated proteins were detected by western blot analysis. *D*, Cells were transfected with either control siRNA or CTR9 siRNA. Approximately 48 hours later, protein levels of CTR9, SPT5, NELF-E, and GAPDH were detected by western blot analysis. *E*, Cells were transfected with the indicated siRNAs and treated with IL-6 and IL-6sR (20 ng/ml each) for the indicated time period. The c-Fos mRNA induction level was measured by RT-qPCR. *E*, NELF-E and SPT5 knockdown efficiencies were measured by RT-qPCR (Left) and western blot analysis (Right). mRNA expression levels were normalized to β-Actin. *F*, Cells were transfected with the indicated siRNAs and treated with IL-6 and IL-6sR (20 ng/ml each) for the indicated time period. Expression levels were normalized to β-Actin. **p<0.01, ***p<0.001 by Student's t test. Error bars represents SD (n = 3).

Our data suggested that CTR9 acts to prevent the transition from transcription initiation to elongation at the *c-Fos* locus. To investigate the molecular mechanism of PAFc regulation of *c-Fos* transcription, we examined the recruitment of elongation factors to the *c-Fos* locus in the presence or absence of PAFc. The negative elongation factor (NELF) complex is known to inhibit elongation of the *c-Fos* gene [37]. Therefore, we examined the association of NELF-E with the *c-Fos* locus using the ChIP assay. Under basal conditions, significant amounts of NELF-E were present only in the TSS region, while almost no signal was detected in the 3′ region. Upon IL-6 stimulation, NELF-E rapidly dissociated from the TSS (Fig. 3*A*). When CTR9 was depleted, the association of NELF with the TSS of *c-Fos* was significantly reduced under basal condition (Fig. 3*A*). Upon IL-6 stimulation, however, NELF association increased to a level seen after activation in control cells. As a result, similar levels of NELF association were achieved in both cases, although they started from different points. It is noteworthy to mention that NELF is known to be recruited to target locus to prevent re-initiation and premature termination [38], [39]. Therefore we postulate that CTR9 might also function to prevent or delay the re-association of NELF to target locus. This result is in agreement with the increased CDK9 (p-TEFb) recruitment observed at the *c-Fos* loci after CTR9 knockdown (Fig. 2*D*). It also explains why RNA Pol II at the *c-Fos* locus was heavily phosphorylated and efficiently preceded in the CTR9-deficient basal conditions (Fig. 2*B and 2C*). Since the recruitment of NELF was changed by CTR9 knockdown, we examined whether CTR9 physically interacts with NELF. CTR9 protein interacted with NELF-E at basal states and this interaction became weaker after IL-6 stimulation (Fig. 3*B*).

We next examined the association of SPT5, a component of the DRB sensitivity inducing (DSIF) complex, with the *c-Fos* locus. Upon depletion of CTR9, more SPT5 was recruited to the TSS region of *c-Fos* after IL-6 stimulation (Fig. 3*C*). However, total protein levels of NELF and SPT5 were not significantly changed by CTR9 knockdown (Fig. 3*D*). Based on these data and our data regarding the association of PAFc with *c-Fos*, we postulated that NELF acts as a negative regulator of *c-Fos* transcription, and that SPT5 acts as a positive regulator of *c-Fos* transcription via PAFc. To test this hypothesis, we determined the levels of *c-Fos* transcripts in NELF-E-depleted and SPT5-depleted cells. In agreement with their physical associations with the locus, the IL-6-induced production of *c-Fos* transcripts was further enhanced in NELF- depleted cells, whereas it was decreased in SPT5-depleted cells (Fig. 3*E*). However, double knockdown of NELF-E or SPT5 along with CTR9 did not amplify the single knockdown effect, indicating that recruitment of NELF or SPT5 and CTR9 are interdependent (Fig. 3*F*). Taken together, these results suggest that PAFc functions to recruit negative elongation factors and block positive elongation factor access to target gene loci.

### PAFc dissociates from the c-Fos locus after IL-6 stimulation

**Figure 4:**
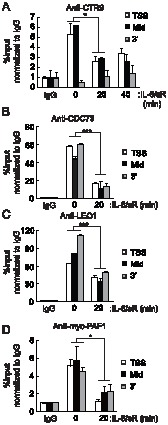
PAFc dissociates from the c-Fos locus after IL-6 stimulation. HepG2 cells were treated with IL-6 plus IL-6sR (20 ng/ml each) for the indicated length of time. Soluble chromatins were immunoprecipitated with anti-CTR9 antibodies, and the bound DNAs were analyzed by quantitative PCR using primers specific to c-Fos (*A*). (*B,C*) CDC73 and LEO1 associations with c-Fos locus were analyzed after IL-6 plus IL-6sR (20 ng/ml each) stimulation. (*D*) Cells were transfected with Myc-PAF1 and 48 hours later, cells were treated with IL-6 plus IL-6sR (20 ng/ml each) for 20 minutes. ChIP assay was performed with anti-Myc antibody and bound DNAs were analyzed by quantitative PCR using primers specific to c-Fos. *p<0.05, ***p<0.001 by Student's t test. Error bars represents SD (n = 3).

To determine whether PAFc affects *c-Fos* transcription directly, we examined the chromatin association patterns of PAFc with the *c-Fos* locus under basal and IL-6 stimulated conditions. CTR9 was primarily associated with the TSS region of *c-Fos* in the absence of stimulation (Fig. 4*A*). Upon stimulation with IL-6, CTR9 rapidly dissociated from the *c-Fos* locus. The IL-6-induced dissociation of CTR9 from the coding region of a gene was not a universal event, because the chromatin association of CTR9 with the coding region of the *Hp* locus was not dramatically altered by the same stimulus (Fig. S2). These results indicate that the association of CTR9 with chromatin is controlled in a locus-specific manner. Like CTR9, CDC73, LEO1, and ectopic myc-PAF1 dissociated from the coding region of the *c-Fos* locus upon IL-6 stimulation (Fig. 4*B–D*). These dissociations are similar to the signal-dependent dissociation of NELF and suggest a possible role of PAFc as a modulator of NELF association with target gene chromatin.

### Dissociation of CTR9 from the c-Fos locus requires signal-activated kinase activity

**Figure 5:**
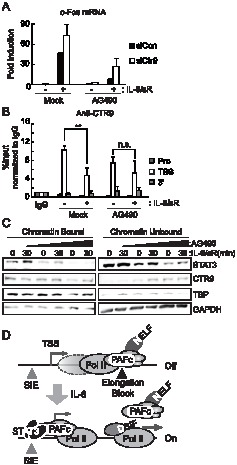
Chromatin association of CTR9 requires kinase activity inhibited by AG490. *A*, Cells were transfected with control siRNA or CTR9 siRNA for 48 hrs and treated with DMSO (mock) or AG490 (100 uM) for 4 hrs prior to IL-6 plus IL-6sR (20 ng/ml each) stimulation for 30 min. The c-Fos mRNA induction level was measured by RT-qPCR. *B*, Cells were treated as in A and a ChIP assay was performed with an anti-CTR9 antibody. Bound DNAs were analyzed by quantitative PCR using primers specific to c-Fos genomic loci. *C*, Cells were treated with DMSO (mock), 100 uM or 200 uM of AG490 for 12 hrs prior to IL-6 plus IL-6sR (20 ng/ml each) stimulation for 30 min. Cells were fractionated into chromatin -unbound and -bound fractions and blotted with anti-CTR9, anti-STAT3, anti-TBP, and anti-GAPDH antibodies. *D*, Negative function of PAFc in IL-6-responsive gene regulation. At TSS of IEG loci in basal condition, PAFc is associated with NELF. Upon IL-6 stimulation, PAFc along with NELF dissociates from the elongation block to allow transcriptional elongation to occur. SIE: Serum Inducible Element (STAT3 binding site). n.s. = not significant, **p<0.01 by Student's t test. Error bars represents SD (n = 3).

Our data collectively indicate that the negative regulation of *c-Fos* transcription by PAFc occurs at the post-initiation step; PAFc must be released from a target locus to proceed to elongation, a process that is induced by IL-6 stimulation. Therefore, we hypothesized that the dissociation of CTR9 from a target locus depends on signaling activity mediated by IL-6. To test this hypothesis, we treated cells with AG490, a kinase inhibitor of JAK2, to inhibit the transcriptional induction of *c-Fos* by IL-6. AG490 significantly reduced IL-6-induced c-Fos expression (Fig. 5*A*). We then measured the association of CTR9 with the *c-Fos* locus in the cells treated with AG490. The association of CTR9 at the *c-Fos* locus was not altered after IL-6 stimulation in AG490-treated cells (Fig. 5*B*). These data suggest that kinase activity related to JAK2 might be responsible for the regulation of the dissociation of CTR9 from the *c-Fos* locus.

To confirm the role of JAK2-related kinase activity in the dissociation of CTR9 from chromatin, we fractionated cells into chromatin-bound and chromatin-unbound fractions and analyzed the chromatin association patterns of STAT3 and CTR9 (Fig. 5*C*). Upon stimulation with IL-6, more STAT3 was observed in the chromatin-bound fraction. The amount of STAT3 in the chromatin-bound fraction gradually decreased with AG-490 treatment. In contrast, less CTR9 was observed in the chromatin-bound fraction. When cells were treated with AG-490, the association of CTR9 with chromatin decreased, and at the same time, more CTR9 appeared in the chromatin-unbound fraction. This result is in complete agreement with the ChIP data obtained from the experiments on the *c-Fos* locus described above (Fig. 5*B*) and implies that the dissociation of CTR9 from chromatin is a dynamically regulated process that requires JAK2-related kinase activity.

## Discussion

Our data suggest that the mammalian PAFc acts as a negative regulator of transcription by aiding recruitment of the negative elongation factor NELF, which blocks the transition of RNA polymerase II-mediated transcription from the initiation to the elongation phase (Fig. 5*D*), to target loci. We observed that phosphorylation of RNA Pol II CTD on serine 5 and serine 2 residues were increased by CTR9 knockdown although the recruitment of RNA Pol II was not increased in unstimulated condition. These data indicate that *c-Fos* transcription relies more heavily on the release of the elongation block than de novo transcription. This finding again emphasizes the importance of elongation control in the transcriptional regulation of immediate early genes.

PAFc was originally identified as a transcriptional activator. However, accumulating evidence suggests that PAFc also has a negative role in gene regulation [23]–[25]. Although a differential effect of H3K4 methylation on different loci has been proposed to explain the dual functions of PAFc [24], it is still not clear how PAFc functions as both a positive and negative factor at the same time. It is well known that PAFc physically interacts with multiple proteins; thus, PAFc might change its interaction partners as transcription proceeds. For example, PAFc recruits H3K4 methyltransferase SET1 to RNA Pol II in the promoter-proximal region, while it recruits SET2, which methylates H3K36, to Pol II in the coding region [19], [20]. It is noteworthy that SET2 recruitment to its target region relies on the phosphorylation status of the RNA Pol II CTD, which is mediated by Ctk1 (a putative P-TEFb homolog in yeast) [40], indicating that P-TEFb may play a role in the SET1/SET2 interactions with PAFc. Here, we showed that recruitment of P-TEFb (CDK9) is also regulated by PAFc at the *c-Fos* locus (Fig. 2*D*).

Originally identified as a negative regulator of elongation, NELF/DSIF also acts as a positive regulator; genome-wide analyses revealed that one-third of NELF associated genes were up-regulated by NELF-depletion, while the remaining two thirds were down-regulated [41]. Similarly, genome-wide analyses of an *Spt5* mutant demonstrated that SPT5 has dual functions in transcription regulation [42]. Although NELF is known to differentially affect chromatin architecture [41], it is not clear how NELF/DSIF performs dual roles, or how specific target genes are selected for positive or negative regulation. Therefore, it will be interesting to investigate how PAFc functions as a modulator of NELF/DSIF-mediated gene regulation. In support of this idea, it has recently been suggested that PAFc aids the molecular function of NELF and DSIF through physical interaction [43].

We observed that PAFc dissociated from target loci locus in response to IL-6 signals, and that this behavior was sensitive to AG490, an inhibitor of JAK2 kinase (Fig. 5*B, C*). Therefore, our data indicate that the phospho-state of PAFc might be dynamically regulated by external stimuli, which play key roles in the regulation of transcription. So far, neither the signal-dependent regulation of PAFc nor the posttranslational modification of PAFc has been explored in detail. Although PAFc is known to interact with RNA Pol II in many of its states, such as the non-phosphorylated, serine 2-phosphorylated, and serine 5-phosphorylated forms [30], [44]–[46], whether PAFc undergoes posttranslational modification during transcriptional processes is unclear. Interestingly, phosphorylation of CDC73 at the tyrosine residue has recently been reported to function in its physical interaction with β-catenin, suggesting that post-translational modifications of PAFc may play important roles in the regulation of transcription [*47*].

We previously reported the function of PAFc as a transcriptional activator in APP gene expression. CTR9 regulated APP gene transcription at promoter region by stabilizing STAT3 association with chromatin. In this study, we have demonstrated a novel function of PAFc controlling the elongation block of the *c-Fos* locus through the regulation of NELF/DSIF recruitment. These two studies suggest a dual function of PAFc in target gene expression. It is interesting to note that although the mode of action is opposite, the end-result is similar. For the transcriptional activation of IL-6-dependent APP gene induction, PAFc is actively recruited to the target locus and aids in gene induction. For the transcriptional activation of IL-6-dependent IEG induction, PAFc is specifically dissociated from the target locus and again, helps gene induction. Therefore it will be interesting to study what determines the locus specificity or specific genomic occupancy of PAFc in the basal condition. Transcriptional potentials of PAFc might be also modulated by its interacting partners, which results in both positive and negative outcomes.

Our results provide new insights that will help improve our understanding of PAFc's mechanism of negative regulation. It will be of great importance to further investigate the signal-dependent regulation of PAFc in relation to its composition and posttranslational modification status to better understand the complexity of the regulation of eukaryotic transcriptional elongation.

## Supporting Information

Figure S1 **Overexpression of PAFc components did not affect** ***c-Fos*** **expression** ***A*** **.** The Myc-CTR9 expression vector was transfected to HepG2 cells. Approximately 48 hours later, cells were treated with IL-6 plus IL-6sR (20 ng/ml each) for the indicated length of time. Left, western blot assay was performed to detect exogenous Myc-CTR9. Right, *c-Fos* mRNA levels were measured by RT-qPCR analysis. Expression levels were normalized to β-Actin. *B*. Cells were transfected with Myc-Paf1 or Myc-Cdc73. 48 hours later, cells were treated with IL-6 plus IL-6sR (20 ng/ml each) for 1 hour and PAF1, CDC73 and GAPDH proteins levels were analyzed by westernblot analysis using endogenous antibodies. *c-Fos* mRNA levels were measured by RT-qPCR analysis.
(EPS)

Figure S2 **Top, diagram of the** ***Hp*** **genomic loci.** PCR primers used for ChIP analyses are shown as two-sided arrows. HepG2 cells were treated with IL-6 plus IL-6sR (20 ng/ml each) for the indicated length of time. Soluble chromatins were immunoprecipitated with anti-CTR9 antibodies, and the bound DNAs were analyzed by quantitative PCR using primers specific to *Hp* loci. Error bars represents SD (n = 3).
(EPS)

Table S1 **Primer sequences used for PCR experiments.**
(DOC)
