# Supplementary material for: CTR9, a Component of PAF Complex, Controls Elongation Block at the c-Fos Locus via Signal-Dependent Regulation of Chromatin-Bound NELF Dissociation
Source: PLoS One. 2013 Apr 11;8(4):e61055. doi: 10.1371/journal.pone.0061055 (PMC3623864; doi:10.1371/journal.pone.0061055)
Supplement: Table S1 — Primer sequences used for PCR experiments. (DOC) [file pone.0061055.s003.doc]

**Supplementary information**

**Supplemental TABLE 1. Primer sequences used for PCR experiments.**

| Primer name | Sequence |
| --- | --- |
| c-Fos unspliced-F | AAGCCCATTCCATCCCAACTCA |
| c-Fos unspliced-R | TCCTTTCCCTTCGGATTCTCCT |
| c-Fos spliced-F | AACTTCATTCCCACGGTCACTG |
| c-Fos spliced-R | TGAGCTGCCAGGATGAACTCTA |
| c-Fos_Pro-F | AGCAGTTCCCGTCAATCCC |
| c-Fos_Pro-R | AGGGCTACAGGGAAAGGC |
| c-Fos_TSS-F | CATCTGCAGCGAGCATC |
| c-Fos_TSS-R | CATGCTGGAGAAGGAGTCT |
| c-Fos_Mid-F | AAGCCCATTCCATCCCAACTCA |
| c-Fos_Mid-R | TCCTTTCCCTTCGGATTCTCCT |
| c-Fos_3'-F | GGACTCAAGTCCTTACCTCT |
| c-Fos_3'-R | CACACTATTGCCAGGAACAC |
| c-Hp_TSS-F | GGTCATAGAGTTGCCAGGTT |
| c-Hp_TSS-R | TATGCTGCCACTAGCTCACT |
| c-Hp_CD-F | AGGGTCACCAAGGGTCTTGTTCAT |
| c-Hp_CD-R | AAGAGCAGAAGAGGCTGGGTCTTT |
| 2.0kb promoter-luc-F | GG GGTACC AGAGCAGCTCCAAGGAATCTGTGT |
| 2.0kb promoter-luc-R | GA AGATCT GAGTGTAAACGTCACGGGCTCAAC |
| 0.4kb promoter -luc-F | GGGGTACC GCA TTG AAC CAG GTG CGA ATG TTC T |
| 0.4kb promoter -luc-R | GAAGATCT GCA TCA CTT GCT TGA AAG GGG GTT TG |
| SV40-0.9kb 1st intron-luc-F | GG GGTACC ATGATGTTCTCGGGCTTCAACGC |
| SV40-0.9kb 1st intron-luc-R | GG GGTACC AGAGCAGCTCCAAGGAATCTGTGT |
| 3.5kb genomic-luc-F | GG GGTACC ATTCATAAAACGCTTGTTATAAAAGCAGT |
| 3.5kb genomic-luc-R | GA AGATCT GGTCGCATTCAACTTAAATGCTTTTATT |
